# Hypertension at diagnosis of coarctation of the aorta as a risk factor for recoarctation

**DOI:** 10.1038/s41390-025-03801-y

**Published:** 2025-01-16

**Authors:** Nofar Berman, Shani Pozailov, Hanna Krymko, Leonel Slanovic, Michael Murninkas, Michael Grunseid, Aviva Levitas

**Affiliations:** 1 Faculty of Health Sciences, Joyce & Irving Goldman Medical School at Ben Gurion University of the Negev, Beer-Sheva, Israel; 2 Department of Pediatrics, Soroka University Medical Center, Beer-Sheva, Israel; 3 Department of Pediatric Cardiology, Soroka University Medical Center, Beer-Sheva, Israel; 4 Clinical Research Center, Soroka University Medical Center, Ben-Gurion University of the Negev, Beer-Sheva, Israel; 5 Medical School for International Health, Ben Gurion University of the Negev, Beer-Sheva, Israel

## Abstract

**Background:**

Coarctation of the aorta (CoA) is a narrowing of the aorta that affects 5–8% of congenital heart defects. Treatment options include surgical repair or transcatheter management with endovascular stenting or balloon dilatation. Late complications after operative repair include systemic hypertension, aortic valve abnormalities, aortic aneurysm, and recoarctation. This study examines the association between the presence of hypertension at the diagnosis of CoA and recoarctation.

**Methods:**

This retrospective study analyzed medical records of patients at Soroka University Medical Center who underwent treatment for CoA between 1978 and 2021. The study included 128 patients diagnosed with CoA and who underwent repair; 9 were excluded, leaving 119 patients for analysis.

**Results:**

Of the 119 patients, 28 developed recoarctation within 15 years of initial repair. Patients with hypertension at initial diagnosis were more likely to develop recoarctation, adjusted to sex and ethnicity. Other patient characteristics were not significantly associated with recoarctation.

**Conclusions:**

Hypertension at the time of initial CoA diagnosis is a risk factor for the development of recoarctation within 15 years of initial repair. Close monitoring and management of blood pressure may be important for patients with CoA. Future research should investigate whether hypertension control can reduce recoarctation risk in this population.

**Impact:**

- Previous studies focus on post-repair hypertension in CoA.
- Limited research on hypertension during CoA diagnosis.
- Knowledge gap on its impact on recoarctation risk.
- Hypertension at CoA diagnosis may predict recoarctation.
- Enables tailored monitoring and timely intervention.

## Introduction

Coarctation of the aorta (CoA) refers to the narrowing or stenosis of the aorta, typically at the aortic isthmus of the proximal thoracic aorta, and may be discrete or a long segment.^1,2^ This narrowing is characterized by localized medial thickening and infolding of the vascular tissue.

The prevalence of CoA varies between 5% and 8% of all congenital heart defects.^2^ Most cases of CoA are sporadic.^1^ CoA typically occurs as an isolated cardiac abnormality. However, patients presenting in infancy are more likely to have associated intracardiac abnormalities.^2^

Most patients present in infancy with shock and heart failure following closure of the ductus arteriosus, on routine screening due to absence of the femoral arterial pulse, or with mild arterial hypertension and show improvement with opening of the ductus arteriosus after initiation of prostaglandin infusion.^2,3^

Hypertension in neonates is normally seen in 0.2–3% of all infants, and because of the low prevalence in otherwise healthy term infants routine blood pressure (BP) determination is not recommended in this age bracket.^4^ Common causes of hypertension in neonates include thromboembolic events and congenital problems such as structural renal malformations, renovascular disease, and CoA.^5^ Hypertension in patients with CoA is likely to be multifactorial and related to a combination of innate abnormalities of the aortic wall and regulatory alterations of renin-angiotensin and baroreceptor systems.^3^

Several surgical and percutaneous treatment techniques for CoA are available including end-to-end resection, patch repair, tube grafts, subclavian flap arterioplasty, balloon dilation, and stenting.^6,7^ The choice of surgical vs endovascular repair is based on diverse factors such as aortic arch anatomy, associated cardiac anomalies, patient preference, and institutional expertise.^8,9^

Apart from the severity of coarctation, long-term survival rates are significantly affected by age at intervention, with the lowest mortality rates observed in patients who underwent surgery between 1 year and 5 years of age, as well as by arterial BP at the first post-operative visit.^10^ Late cardiovascular complications after operative repair of CoA include systemic hypertension, premature coronary artery disease, aortic valve abnormalities, aortic aneurysm, and recoarctation. Recoarctation is reported to occur after successful operative repair in 3–26% of survivors.^10^

In addition, CoA has been associated with an increased risk of intracranial aneurysms (IA) and their rupture, which occur at an early age compared with patients without CoA. To explain this association, there are two main etiopathologic factors- the first is vascular developmental abnormality and the second is that arterial hypertension is a main risk factor for the development of these aneurysms.^11,12^

Previous studies have shown that systemic arterial hypertension following coarctation repair is common and often observed even in patients with a successful CoA repair and is associated with significant morbidity and early mortality. It is postulated that there is a residual narrowing of the aorta in addition to dysfunction of the normal control mechanisms regulating BP during growth and development in patients with repaired CoA.^3,8,9,13,14^ In our study, we examined the association between the presence of hypertension at the diagnosis of CoA and the clinical outcomes of these patients, and explored the possibility that hypertension at the initial presentation of CoA could be indicative of the prognosis of CoA patients. The findings have the potential to change the routine follow-up after patients with HTN following their first diagnosis of CoA, with close monitoring, magnetic resonance imaging from an early age for IA diagnosis, and home BP measurements.

## Methods

This study was designed as a retrospective population-based cohort study to examine the association between the presence of hypertension at the diagnosis of CoA and recoarctation and mortality. The study population consisted of all patients diagnosed with CoA at Soroka University Medical Center (SUMC) in southern Israel between 1978 and 2021, in all 143 patients. Patients who underwent repair of CoA were included in the study group (*n* = 128). Patients who died during the first month following the index procedure, patients with missing data on their initial presentation on diagnosis, and patients with specific complexed heart defects were excluded (*n* = 9).

The study protocol involved comparing the exposed group (patients with hypertension during CoA diagnosis) to the unexposed group (patients without hypertension during CoA diagnosis) in terms of primary and secondary outcomes. The primary outcome was recoarctation, and secondary outcomes included time to recoarctation and all-cause 15-year mortality. Data analysis was performed using R studio (version 1.2.5033), and statistical tests and/or confidence intervals were performed at *P* = 0.05 (two-sided) except for those specified otherwise.

Descriptive statistics were provided using summary tables, and statistical tests were used for analysis: student *t*-test for numeric variables normally distributed, Mann–Whitney test for a-parametric numeric variables, and Pearson’s χ^2^ test for binary variables. Survival analysis was performed using Kaplan–Meier methodology and the Cox-proportional hazard model while adjusting to sex and ethnicity. All *p*-values were reported rounded to three decimal places, and percentages were rounded to one decimal place. This study was conducted in accordance with the principles of the Declaration of Helsinki and was approved by the Ethics Committee of SUMC in Beer-Sheva. The confidentiality and privacy of all participants were strictly protected throughout the study.

## Results

The two groups of patients with and without HTN during diagnosis of CoA were similar in their clinical, sociodemographic, echocardiographic, and electrocardiogram characteristics.

Although there were no statistically significant differences, the HTN group in our study included a higher proportion of female patients (42.4% vs 30.2%) and patients of Jewish origin (54.5% vs 41.9%) compared to the non-HTN group. The median age at diagnosis was significantly higher in the non-HTN group (0.13 months vs 0.6, *p* = 0.002). There were no statistically significant differences between the two groups in terms of the need for drug administration at the time of diagnosis, congestive heart failure (CHF), shock, or ICU admission (Table 1).

**Table 1:** Univariate analysis of clinical and sociodemographic characteristics.

	Non-hypertension (*N* = 86)	Hypertension (*N* = 33)	*p*-value
Male, *n*/*N* (%)	60 (69.8)	19 (57.6)	0.297
Bedouin, *n*/*N* (%)	50 (58.1)	15 (45.5)	0.299
Age at diagnosis, month			
Mean ± SD (*n*)	12.91 ± 36.51	48.48 ± 77.34	
Median	0.6	0.13	0.002
Min; Mmax	0.00; 242.43	0.00; 221.37	
Need for drug administration for coarctation diagnosis, *n*/*N* (%)	56 (65.1)	21 (63.6)	1
CHF at diagnosis, *n*/*N* (%)	35 (40.7)	11 (33.3)	0.597
Shock at diagnosis, *n*/*N* (%)	32 (37.2)	9 (27.3)	0.42
ICCU^a^ at diagnosis, *n*/*N* (%)	66 (76.7)	19 (57.6)	0.065

^a^Intensive coronary care unit

There was no significant difference in the prevalence of ASD, VSD, PDA, PFO, Shone complex, or the type of native coarctation between the two groups. However, there was a significant difference in the prevalence of an interrupted arch in the HTN group (9.1% vs 0%, *p* = 0.013), as one of the clinical signs of this disease is hypertension (Table S1).

Reviewing the interventional characteristics of the two groups, the first procedure type is significantly different between the two groups, with a higher proportion of surgical repair in the non-HTN group (96.5% vs 72.7%) and a higher proportion of balloon dilation and stent in the HTN group (27.3% vs 3.5%). The time from diagnosis to first procedure is also significantly different between the two groups (*p* = 0.016) with a longer time in the HTN group (23 vs 10 days) (Table 2).

**Table 2:** Univariate analysis of interventional characteristics.

	Non-hypertension (*N* = 86)	Hypertension (*N* = 33)	*p*-value
First procedure type, *n*/*N* (%)			0.001
Surgery, *n*/*N* (%)	83 (96.5)	24 (72.7)	
Balloon dilatation, *n*/*N* (%)	1 (1.2)	4 (12.1)	
Stent, *n*/*N* (%)	2 (2.3)	5 (15.2)	
Days from diagnosis to first procedure^a^ (median [IQR])	10.00 [4.50, 32.50]	23.00 [15.00, 99.75]	0.016
Surgical repair (%)	84 (97.7)	25 (75.8)	<0.001

^a^First procedure for CoA repair—including surgery/balloon dilatation/stent.

The proportion of patients with 15-year recoarctation is significantly higher in the HTN group (*p* = 0.022) and there is no significant difference in the proportion of 15-year mortality between the two groups (Table 3).

**Table 3:** Outcomes.

	Non-hypertension (*N* = 86)	Hypertension (*N* = 33)	*p*-value
Time from first procedure to recoarctation, months (median [IQR])	28.60 [5.87, 108.17]	77.73 [39.61, 149.44]	0.18
15-year recoarctation, *n*/*N* (%)	15 (17.4)	13 (39.4)	0.022
15-year mortality, *n*/*N* (%)	7 (8.1)	0 (0.0)	0.210

When examining the association between the outcome of recoarctation and various risk factors, such as the type of coarctation, the type of CoA repair performed (surgery vs stent and balloon dilation), the age at diagnosis, and the time from diagnosis to the initial procedure, none of them is statistically significant (Table S2).

Although both, age at diagnosis and the type of CoA repair initially appeared to show significant differences between the HTN and non-HTN groups when comparing baseline characteristics, neither age at diagnosis (HR 1.00, 95% CI: 0.99–1.01) nor surgical repair (HR 0.64, 95% CI: 0.18–2.27) demonstrated a significant impact on the risk of recoarctation within 15 years when these variables were incorporated into the multivariate Cox regression analysis (Table S4). HTN at diagnosis of coarctation was a statistically significant risk factor for recoarctation within 15 years following the first coarctation repair in the univariate Cox-proportional hazard analysis (HR 2.31, 95% CI: 1.099–4.858) (Table S3).

**Fig. 1:**
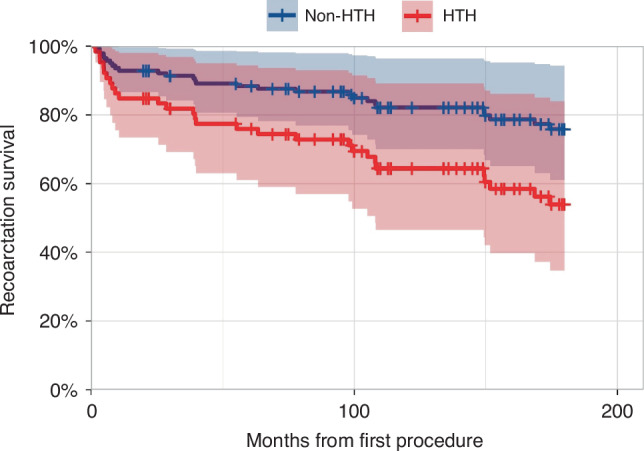
Survival graph by Cox Multivariate model for 15 years recoarctation by hypertension. The red curve represents patients with hypertension (HTN), while the blue curve represents patients without hypertension (non-HTN). Shaded areas indicate 95% confidence intervals for each group. The plot illustrates a higher recoarctation rate in the HTN group compared to the Non-HTN group over the 15-year follow-up period.

Moreover, when adjusting for demographic covariates (sex and ethnicity), only HTN remained statistically significant (HR 2.24, 95% CI: 1.05–4.77) (Table 4 and Fig. 1).

**Table 4:** Results of a multivariate Cox regression to 15 years recoarctation with HTN and factors associated with mortality.

	HR	95% CI
Hypertension	2.24	1.05, 4.77
Sex (male)	0.80	0.37, 1.72
Ethnicity (Bedouin)	1.02	0.48, 2.18

*HR* hazard ratio, *CI* confidence Interval.

## Discussion

CoA is a complex cardiovascular condition that requires prompt diagnosis and appropriate management. Previous studies have provided valuable insights into the association between hypertension after CoA repair and clinical outcomes in these patients.

It is assumed that hypertension in patients with CoA likely stems from multiple biological mechanisms, such as innate morphological abnormalities of the aortic wall, residual pressure gradients following repair, and dysregulation of the renin-angiotensin and baroreceptor systems. Residual narrowing or hypoplasia of the aortic arch post-repair can cause regional hypertension in the arch and its branches, while recoarctation at previously repaired sites further elevates BP in pre-coarctation arterial segments.^8,9,13^ Evidence suggests that even after successful stenting of coarctation, BP often fails to normalize. Furthermore, renal hypoperfusion and ischemia may activate the renin–angiotensin–aldosterone system, contributing to persistent hypertension even after CoA repair. Longstanding hypertension may also reset the carotid and aortic baroreceptors to higher pressure thresholds, impairing BP regulation in these patients.^3^

These patients suffer from increased cardiovascular morbidity and mortality, which is mostly attributed to the increased prevalence of hypertension contributing to long-term complications in CoA patients.^14^

However, there remained a need to further explore the clinical characteristics of patients with CoA at the time of diagnosis and their impact on long-term outcomes.

In this study, we aimed to fill this knowledge gap and found that hypertension during the diagnosis of CoA is a significant risk factor for poor clinical outcomes in these patients, with increased rates of recoarctation (Fig. 1).

The results of our study may have important implications for the management of patients with CoA. This is in line with previous research that indicated a link between CoA and the development of IA, which may be due to a combination of vascular developmental abnormalities and HTN as a risk factor.^11,12^

One strength of our study was its physical setting at Soroka University Medical Center which provides medical services to a population of over a million people and has the highest number of births in Israel (~18,000 births a year), which increases sample generalizability.

One limitation of our study is the potential influence of age at diagnosis and the type of CoA repair on long-term outcomes. While these variables showed significant differences between the HTN and non-HTN groups at baseline, our analysis found no significant association between them and recoarctation. However, it is possible that other confounding factors may have overshadowed their effects.

Another limitation of our study was its relatively small sample size which may have been insufficient to detect subtle but clinically meaningful differences. Due to the rarity of CoA, parallel studies on this topic present similar or smaller sample sizes. Larger, multi-center studies or meta-analyses will be required to provide more definitive insights into the factors that influence long-term outcomes in CoA. Furthermore, more research must be done to elucidate the pathophysiological connection between HTN at the time of diagnosis and recoarctation after repair.

## Conclusions

Our findings suggest that HTN at the diagnosis of CoA should be considered as a predictor of poor clinical outcomes.

While this study sheds some light on potential risk factors for recoarctation in patients with CoA, the results should be interpreted with caution. It may be warranted to consider close monitoring and home BP measurements in these patients but further research with larger sample sizes and a more comprehensive assessment of potential risk factors is needed to better understand the predictors of recoarctation and how they can be managed. Additionally, it would be valuable to explore the impact of different treatment strategies for hypertension on the outcomes of patients with CoA, in order to optimize their management.

## Supplementary information

Supplementary information

## Data Availability

The datasets generated during and/or analyzed during the current study are available from the corresponding author upon reasonable request.

## References

[1] Rao, P. S. Coarctation of the aorta. *Curr. Cardiol. Rep.* **7**, 425–434 (2005). 16256011 10.1007/s11886-005-0060-0

[2] Ganigara, M. et al. Preoperative physiology, imaging, and management of coarctation of aorta in children. *Semin Cardiothorac. Vasc. Anesth.* **23**, 379–386 (2019). 31535945 10.1177/1089253219873004

[3] Vigneswaran, T. V., Sinha, M. D., Valverde, I., Simpson, J. M. & Charakida, M. Hypertension in coarctation of the aorta: challenges in diagnosis in children. *Pediatr. Cardiol.* **39**, 1–10 (2018). 29043396 10.1007/s00246-017-1739-x

[4] Dionne, J. M., Abitbol, C. L. & Flynn, J. T. Hypertension in infancy: diagnosis, management and outcome. *Pediatr. Nephrol.* **27**, 17–32 (2012). 21258818 10.1007/s00467-010-1755-z

[5] Flynn, J. T. Neonatal hypertension: diagnosis and management. *Pediatr. Nephrol.* **14**, 332–341 (2000). 10775081 10.1007/s004670050771

[6] Tanous, D., Bensona, L. N. & Horlick, E. M. Coarctation of the aorta: evaluation and management. *Curr. Opin. Cardiol.* **24**, 509–515 (2009). 19667980 10.1097/HCO.0b013e328330cc22

[7] Martins, J. D. et al. Impact of treatment modality on vascular function in coarctation of the aorta: the LOVE-COARCT study. *J. Am. Heart Assoc.* **8**, e011536 (2019). 30929556 10.1161/JAHA.118.011536 PMC6509735

[8] Egbe, A. C. et al. Persistent hypertension and left ventricular hypertrophy after repair of native coarctation of aorta in adults. *Hypertension* **78**, 672–680 (2021). 34247510 10.1161/HYPERTENSIONAHA.121.17515 PMC8363521

[9] Kenny, D., Polson, J. W., Martin, R. P., Paton, J. F. R. & Wolf, A. R. Hypertension and coarctation of the aorta: an inevitable consequence of developmental pathophysiology. *Hypertension Res.* **34**, 543–547 (2011). 10.1038/hr.2011.22 21412243

[10] Toro-Salazar, O. H. et al. Long-term follow-up of patients after coarctation of the aorta repair. *Am. J. Cardiol.* **89**, 541–547 (2002). 11867038 10.1016/s0002-9149(01)02293-7

[11] Donti, A. et al. Frequency of intracranial aneurysms determined by magnetic resonance angiography in children (mean age 16) having operative or endovascular treatment of coarctation of the aorta (mean age 3). *Am. J. Cardiol.* **116**, 630–633 (2015). 26096998 10.1016/j.amjcard.2015.05.030

[12] Curtis, S. L. et al. Results of screening for intracranial aneurysms in patients with coarctation of the aorta. *Am. J. Neuroradiol.* **33**, 1182–1186 (2012). 22322607 10.3174/ajnr.A2915 PMC8013223

[13] Canniffe, C., Ou, P., Walsh, K., Bonnet, D. & Celermajer, D. Hypertension after repair of aortic coarctation—a systematic review. *Int J. Cardiol.* **167**, 2456–2461 (2013). 23041096 10.1016/j.ijcard.2012.09.084

[14] Panzer, J., Bové, T., Vandekerckhove, K. & De Wolf, D. Hypertension after coarctation repair—a systematic review. *Transl. Pediatr.* **11**, 270–279 (2022). 35282025 10.21037/tp-21-418 PMC8905104

