# Supplementary material for: Hypertension at diagnosis of coarctation of the aorta as a risk factor for recoarctation
Source: Pediatr Res. 2025 Jan 16;98(1):237–40. doi: 10.1038/s41390-025-03801-y (PMC12411270; doi:10.1038/s41390-025-03801-y)
Supplement: Supplementary file 1 — Supplementary information [file 41390_2025_3801_MOESM1_ESM.pdf]

Supplements:

Table S1 -Univariate analysis of echocardiograph and electrocardiogram characteristics

	Non-Hypertension (N=86)	Hypertension (N=33)	p-value
ASD, n/N (%)	20 (23.3)	7 (21.2)	1.000
VSD, n/N (%)	51 (59.3)	14 (42.4)	0.147
Aortic Valve, n/N (%)			0.393
Normal, n/N (%)	30 (34.9)	16 (48.5)	
Bicuspid, n/N (%)	49 (57.0)	15 (45.5)	
Other[†], n/N (%)	7 (8.1)	2 (6.1)	
Other valve defect[‡], n/N (%)	20 (23.3)	7 (21.2)	1.000
PDA, n/N (%)	14 (16.3)	3 (9.1)	0.477
PFO, n/N (%)	7 (8.1)	2 (6.1)	1.000
Shone complex, n/N (%)	6 (7.0)	2 (6.1)	1.000
Arch[♀], n/N (%)			0.013
Left, n/N (%)	84 (97.7)	30 (90.9)	
Double, n/N (%)	2 (2.3)	0 (0.0)	
Interrupted, n/N (%)	0 (0.0)	3 (9.1)	
Pathological ECG, n/N (%)	33 (41.2)	15 (57.7)	0.216
AXIS[˫], n/N (%)			0.339
Normal, n/N (%)	73 (91.2)	22 (84.6)	
LAD, n/N (%)	5 (6.2)	2 (7.7)	
RAD, n/N (%)	2 (2.5)	1 (3.8)	
Extreme, n/N (%)	0 (0.0)	1 (3.8)	
VH[ϖ], n/N (%)			0.325
Normal, n/N (%)	71 (88.8)	21 (80.8)	
LVH, n/N (%)	6 (7.5)	2 (7.7)	
RVH, n/N (%)	3 (3.8)	3 (11.5)	
TOC[ж], n/N (%)			0.055
Juxta-ductal, n/N (%)	74 (86.0)	23 (69.7)	
Long segment, n/N (%)	12 (14.0)	9 (27.3)	
Abdominal aorta, n/N (%)	0 (0.0)	1 (3.0)	

ASD = Atrial Septal Defect, VSD = Ventricular Septal Defect, PDA = Patent Ductus Arteriosus, PFO = Patent Foramen Ovale, LAD = Left Axis Deviation, RAD = Right Axis Deviation, Extreme = Extreme Axis Deviation, LVH = Left Ventricular Hypertrophy, RVH = Right Ventricular Hypertrophy

† Other aortic valve defects including aortic stenosis and regurgitation

‡ Mitral, tricuspid and pulmonary valve defects

♀ Type of aortic arch

Ͱ ECG AXIS

ϖ Ventricular Hypertrophy sign on ECG

Ж Type of native Coarctation

Table S2- Association between outcome (recoarctation) and risk factors

	15 year Non-recoarctation (N=91)	15 year Recoarctation (N=28)	p-value
Arch, n/N (%)			0.153
Left, n/N (%)	88 (96.7)	26 (92.9)	
Double, n/N (%)	2 (2.2)	0 (0.0)	
Interrupted, n/N (%)	1 (1.1)	2 (7.1)	
TOC, n/N (%)			0.184
Juxta-ductal, n/N (%)	76 (83.5)	21 (75.0)	
Long segment, n/N (%)	15 (16.5)	6 (21.4)	
Abdominal aorta, n/N (%)	0 (0.0)	1 (3.6)	
Pathological ECG, n/N (%)	34 (42.0)	14 (56.0)	0.317
First procedure type, n/N (%)			0.133
Surgery, n/N (%)	84 (92.3)	23 (82.1)	
Balloon dilatation, n/N (%)	2 (2.2)	3 (10.7)	
Stent, n/N (%)	5 (5.5)	2 (7.1)	
Days from diagnosis to first procedure (median [IQR])	12.50 [5.00, 41.50]	17.00 [9.00, 208.00]	0.118
Surgical repair, n/N (%)	85 (93.4)	24 (85.7)	0.372
Age at diagnosis, month<br>Mean ±SD (n)<br>Median<br>Min; Max	 19.19±50.36<br>0.20<br>0.00; 221.37	 35.51±62.52<br>1.80<br>0.00; 242.43	0.186

Table S3- Results of a univariate Cox regression to 15 years recoarctation

	HR	95% CI
HTN	2.31	1.09, 4.85
Sex (Male)	0.711	0.34, 1.50
Ethnicity (Bedouin)	0.901	0.43, 1.89

HR = Hazard Ratio, CI = Confidence Interval

Table S4- Results of a multivariate Cox regression to 15 years recoarctation

	HR	95% CI
HTN	2.70	1.13, 6.47
Sex (Male)	1.13	0.48, 2.64
Ethnicity (Bedouin)	0.90	0.39, 2.04
Age at Diagnosis (months)	1.00	0.99, 1.01
Surgical Repair (%)	0.64	0.18, 2.27

HR = Hazard Ratio, CI = Confidence Interval
